# Safety and tolerability of quizartinib, a FLT3 inhibitor, in advanced solid tumors: a phase 1 dose-escalation trial

**DOI:** 10.1186/s12885-018-4692-z

**Published:** 2018-08-06

**Authors:** Kyriakos P. Papadopoulos, Eytan Ben-Ami, Amita Patnaik, Denise Trone, Jianke Li, George D. Demetri

**Affiliations:** 1 South Texas Accelerated Research Therapeutics, 4383 Medical Dr, Suite 4021, San Antonio, TX 78229 USA; 2 Dana-Farber Cancer Institute, 450 Brookline Ave, Boston, MA 02215 USA; 3 Formerly Daiichi Sankyo Pharma Development, 3172 Mount Acmar Court, San Diego, CA 92111 USA; 4 Daiichi Sankyo Pharma Development, 10201 Wateridge Circle, Suite 240, San Diego, CA 92121 USA; 5 Ludwig Center at Harvard, Harvard Medical School, 450 Brookline Ave, Boston, MA 02215 USA

**Keywords:** Quizartinib, Receptor tyrosine kinase inhibitor, FLT3, PDGFR

## Abstract

**Background:**

Quizartinib, an inhibitor of class III receptor tyrosine kinases (RTKs), is currently in phase 3 development for the treatment of acute myeloid leukemia (AML) bearing internal tandem duplications in the *FLT3* gene. Aberrant RTK signaling is implicated in the pathogenesis of a variety of solid tumors, suggesting that inhibiting quizartinib-sensitive RTKs may be beneficial in precision cancer therapy.

**Methods:**

This was a phase 1, open-label, modified Fibonacci dose-escalation study of orally administered quizartinib in patients with advanced solid tumors whose disease progressed despite standard therapy or for which there was no available standard treatment. Patients received quizartinib dihydrochloride (henceforth referred to as quizartinib) once daily throughout a 28-day treatment cycle. The primary endpoint was evaluation of the maximum tolerated dose (MTD) of quizartinib. Secondary endpoints included preliminary evidence of antitumor activity and determination of the pharmacokinetic and pharmacodynamic parameters of quizartinib.

**Results:**

Thirteen patients were enrolled. Five patients received a starting dose of quizartinib 135 mg/day; dose-limiting toxicities (DLTs) of grade 3 pancytopenia, asymptomatic grade 3 QTc prolongation, and febrile neutropenia were observed in 1 patient each at this dose. A lower dose of quizartinib (90 mg/day [*n* = 8]) was administered without DLTs. The most common treatment-related treatment-emergent adverse events (AEs) were fatigue (*n* = 7, 54%), dysgeusia (*n* = 5, 38%), neutropenia (*n* = 3, 23%), and QTc prolongation (*n* = 3, 23%). Overall, all patients experienced at least 1 AE, and 4 experienced serious AEs (2 patients each in the 135-mg and 90-mg dose groups) including hematologic AEs, infections, and gastrointestinal disorders. Six patients (including 3 patients with gastrointestinal stromal tumors [GIST]) had a best response of stable disease.

**Conclusion:**

The MTD of quizartinib in patients with advanced solid tumors was 90 mg/day. Overall, the safety and tolerability of quizartinib were manageable, with no unexpected AEs. Quizartinib monotherapy had limited evidence of activity in this small group of patients with advanced solid tumors.

**Trial registration:**

Clinical Trials Registration Number: NCT01049893; First Posted: January 15, 2010.

**Electronic supplementary material:**

The online version of this article (10.1186/s12885-018-4692-z) contains supplementary material, which is available to authorized users.

## Background

Activation of oncogenes as a result of mutations, gene amplifications, or translocations (chromosomal rearrangements) is a key mechanism of disrupting physiologic regulation of cell growth and differentiation [1]. These genetic changes may result in aberrant receptor tyrosine kinase (RTK) activation and signaling, promoting cell proliferation, differentiation and angiogenesis, which contribute to the pathogenesis of uncontrolled tumor growth [2, 3]. Dysregulated RTK signaling, such as KIT or platelet-derived growth factor receptor (PDGFR) alpha overactivation in gastrointestinal (GI) stromal tumors (GIST) [4, 5] or RET activation in thyroid tumors [6], has been observed across a broad spectrum of solid tumors [7–12] and is implicated in both tumorigenesis and cancer progression.

Inhibition of dysregulated RTK signaling by disruption of specific targets in the cancerous cells has proven efficacious in a wide range of malignancies [13]. Though the clinical success of imatinib for the treatment of chronic myelogenous leukemia (CML) and GIST is considered one of the hallmarks of targeted therapy development in cancer care, numerous RTK inhibitors have been approved over the past decade for various malignancies. Notable examples include the epidermal growth factor receptor (EGFR) inhibitors erlotinib and gefitinib for the treatment of advanced EGFR-mutated non-small cell lung cancer; the BRAF and MEK inhibitors vemurafenib and trametinib for metastatic V600-mutated melanomas; and the multi-kinase inhibitor cabozantinib for metastatic medullary thyroid cancer and renal cell carcinoma [14]. Nevertheless, in vitro and clinical evidence indicates that treatment with RTK inhibitors is almost inevitably associated with acquired modifications in the cancerous cells, eventually leading to treatment resistance [15, 16]. Common mechanisms of resistance include point mutations within the kinase domain (decreasing the binding affinity of the RTK inhibitors), modifications of gene copy number and RTK expression levels, modification of signaling pathways, and resistance related to drug influx/efflux (multidrug resistance). The emergence of acquired resistance has led to the investigation of different tyrosine kinase inhibitors (TKIs), based on their kinase affinities, in an attempt to counter these resistance mechanisms.

Quizartinib dihydrochloride (henceforth referred to as quizartinib) is an oral, highly potent, and selective, next-generation FMS-like tyrosine kinase 3 (FLT3) inhibitor [17]. Quizartinib also has affinities, albeit to a lesser extent, for KIT, colony-stimulating factor 1 receptor (CSF1R), RET, and PDFGR alpha and beta (PDGFRA and PDGFRB). These affinities are within 10-fold of quizartinib’s binding affinity for FLT3, but quizartinib has little or no activity against other kinases or non-kinase enzymes, receptors, or channels [18]. Early phase 1 quizartinib studies demonstrated a manageable safety profile, favorable pharmacodynamic activity, and encouraging clinical activity in patients with leukemia [19–21]. Quizartinib is also well tolerated in healthy subjects [22]. Quizartinib also has shown promising activity in relapsed/refractory (RR) acute myeloid leukemia (AML) with *FLT3*-internal tandem duplication (ITD) in phase 1 and 2 studies [23–26] and is currently being evaluated in phase 3 studies in both newly diagnosed and R/R *FLT3*-ITD AML (NCT02668653 and NCT02039726, respectively).

Although kinase affinity data are consistent with inhibition of KIT, PDGFRA, and PDGFRB, the effect of quizartinib on these RTKs at therapeutic doses for patients with advanced solid malignancies is yet to be elucidated. Furthermore, *KIT* mutations are implicated in acquired resistance to imatinib, and the ability of quizartinib to inhibit kinase activity of these KIT variants is unknown. Because preclinical data suggest that quizartinib may inhibit the activity of several RTKs implicated in the pathogenesis of solid tumors, we undertook this phase 1 dose-finding study to evaluate the safety, tolerability, and preliminary antitumor activity of oral quizartinib in patients with advanced solid tumors.

## Methods

### Patients

Eligible patients were ≥ 18 years old with Eastern Cooperative Oncology Group (ECOG) performance status 0–2 and histologically confirmed advanced solid tumors. Patients were required to have at least 1 measurable lesion (by computed tomography or magnetic resonance imaging) according to Response Evaluation Criteria in Solid Tumors (RECIST, version 1.0) [27] that had progressed during or following currently available standard therapy or for which no curative therapy existed. Patients were required to be at least 4 weeks between the last systemic anticancer therapy, immunotherapy, or radiotherapy and the start of study treatment (for patients with GIST receiving an approved TKI, at least 2 weeks since the last dose of that inhibitor) and to have adequate bone marrow, renal, and hepatic function. Exclusion criteria included uncontrolled central nervous system metastases, significant liver or cardiovascular disease (including prolonged corrected QT interval [QTc] ≥ 450 msec in the screening electrocardiograms [ECGs]), and use of drugs known to prolong QTc interval or cytochrome P450 3A (CYP3A) inhibitors. All institutional review boards approved the protocol, and patients provided written informed consent and indicated availability for periodic follow-up at the study site.

### Study design and treatment

This was a phase 1 study using a modified Fibonacci design of intercohort 3 + 3 dose escalation. The treatment consisted of quizartinib once daily as an oral solution without food (1 h prior to or 2 h after a meal) throughout a 28-day treatment cycle. The study was designed to include a maximum of 6 quizartinib dose groups, starting at 135 mg/day and escalating to 700 mg/day. No intrapatient dose escalation was allowed. The starting quizartinib dose of 135 mg/day was based on 1 dose level below the 200-mg daily maximum tolerated dose (MTD) determined in a previous phase 1 study in patients with R/R AML [23]. The first cohort was to enroll at least 3 patients, with dose escalations for subsequent patient cohorts to commence when the third fully evaluable patient in the prior cohort had completed the 28-day dosing regimen with no evidence of dose-limiting toxicity (DLT). If there was only 1 occurrence of DLT in a group of 3 patients, the group was to be expanded to 6 patients. The dose was then to be escalated when the sixth patient had completed 28 days of treatment and there was no more than 1 occurrence of DLT. If > 1 DLT occurred at the starting dose of 135 mg/day, the next group of patients enrolled were to receive a dose of 90 mg/day. If an unacceptably toxic dose level was identified (ie, with ≥2 DLTs), the next-lower dose level proven to be safe and well tolerated would be judged to be the MTD. Once the MTD was determined, additional patients (dose-expansion cohort) enriched for cancers that are pathophysiologically dependent on KIT or PDGFR (such as GIST or melanoma) were to be enrolled to obtain further safety and tolerability data, as well as preliminary indications of potential antitumor activity.

Sample size was planned on the basis of dose escalation, with a target enrollment of between 6 and 45 patients. Patients were discontinued from study drug dosing if they experienced unacceptable toxicity, if the investigator or the patient believed that it was in the patient’s best interest to discontinue study drug dosing, or for disease progression.

### Objectives

The primary objectives of this study were to determine the safety, tolerability, MTD, and recommended phase 2 dosing regimen of quizartinib given once daily, continuously for 28 days (treatment cycle), in patients with advanced solid tumors. The secondary objectives were to investigate the pharmacokinetics (PK) and pharmacodynamic parameters of quizartinib and to assess any preliminary evidence of clinical antitumor activity.

### Assessments

Assessments were scheduled during and after treatment (30 days after the last protocol treatment) with quizartinib for the identification and evaluation of adverse events (AEs) and serious adverse events (SAEs). Physical examinations, vital sign measurements, determination of ECOG performance status, 12-lead ECGs, blood samples, and urinalyses were scheduled at regular intervals (Additional file 1).

Dose-limiting toxicities were defined as grade 4 neutropenia (absolute neutrophil count < 0.5 × 10^9^ cells/L) for 5 or more consecutive days, or grade 3 or 4 neutropenia of any duration with sepsis or a fever greater than 38.5 °C; thrombocytopenia ≤25 × 10^9^ cells/L or bleeding requiring platelet transfusion; grade 3 or 4 nausea, vomiting, or diarrhea despite the use of adequate/maximal medical intervention and/or prophylaxis; other grade ≥ 3 nonhematologic toxicities; left ventricular ejection fraction (LVEF) below lower limit of normal or a 25% decline in LVEF from baseline; and grade ≥ 3 prolongation in QTc (≥ 501 msec on at least 2 separate ECGs) as defined by National Cancer Institute Common Terminology Criteria for Adverse Events (NCI CTCAE) version 4.0.

For assessment of potential antitumor activity, evaluations of target lesions using unidimensional tumor measurements were performed within 28 days prior to study drug administration, on Day 1 of Cycle 2 (± 3 days), and on Day 1 (± 3 days) every 2 cycles thereafter. Target lesions were evaluated using RECIST v1.0.

## Results

Between January 2010 and November 2011, 13 patients were enrolled and received at least 1 dose of quizartinib. Median age at registration was 50.0 years (range, 26–75 years), and 61.5% of patients were female. All patients had ECOG performance status of 0 or 1. Tumor types included GIST (*n* = 3), other subtypes of sarcoma (*n* = 3), colorectal cancer (*n* = 2), thyroid cancer (*n* = 2), melanoma (*n* = 1), gall bladder cancer (*n* = 1), and unknown primary tumor (*n* = 1). All patients had tumor progression following at least 2 prior lines of therapy with a median of 5 prior therapies (range, 2–10); median duration of prior therapies was 2.7 years (range, 0.2–7.6 years). Of the 13 patients, 5 received the 135-mg/day dose and 8 patients subsequently received the 90-mg/day dose (Table 1). Of the 5 patients in the 135-mg dose group, 4 received ≤1 cycle of quizartinib and 1 received 2 cycles. Of the 8 patients in the 90-mg dose group, 7 received ≤1 cycle of quizartinib and 1 received 2 cycles.

**Table 1:** Patient demographics

	Treatment group, 90 mg (*n* = 8)	Treatment group, 135 mg (*n* = 5)	Total (*N* = 13)
Age, years			
Mean (SD)	50.3 (16.05)	53.6 (9.56)	51.5 (13.55)
Median	49.5	56.0	50.0
Min, Max	26, 75	43, 66	26, 75
Age category, *n* (%)			
18–60 years	6 (75.0)	4 (80.0)	10 (76.9)
61–75 years	2 (25.0)	1 (20.0)	3 (23.1)
Male, *n* (%)	3 (37.5)	2 (40.0)	5 (38.5)
Female, *n* (%)	5 (62.5)	3 (60.0)	8 (61.5)
Race, *n* (%)			
White	7 (87.5)	4 (80.0)	11 (84.6)
Asian	0	1 (20.0)	1 (7.7)
Missing	1 (12.5)	0	1 (7.7)
Ethnicity, *n* (%)			
Hispanic or Latino	2 (25.0)	2 (40.0)	4 (30.8)
Not Hispanic or Latino	6 (75.0)	3 (60.0)	9 (69.2)
ECOG performance status, Cycle 1/Day 1, *n* (%)			
0	4 (50.0)	2 (40.0)	6 (46.2)
1	4 (50.0)	3 (60.0)	7 (53.8)
Prior chemotherapy, *n* (%)	5 (62.5)	4 (80.0)	9 (69.2)
Prior TKI therapy, *n* (%)	4 (50.0)	3 (60.0)	7 (53.8)
Tumor type, *n*			
GIST	3	0	3
Sarcoma	2	1	3
Thyroid cancer	1	1	2
Colorectal cancer	1	1	2
Gall bladder cancer	0	1	1
Melanoma	1	0	1
Unknown primary tumor	0	1	1

*ECOG* Eastern Cooperative Oncology Group, *GIST* gastrointestinal stromal tumor, *SD* standard deviation, *TKI* tyrosine kinase inhibitor

All patients had discontinued the study at the time of data cut-off: 9 patients due to progressive disease, 3 on account of investigator/patient choice, and 1 due to an AE (QTc prolongation).

### Safety and tolerability results

All patients received at least 1 dose of study drug and were included in the safety analysis (*N* = 13). Dose-limiting toxicities were observed only in the 135-mg dose group: grade 3 pancytopenia, asymptomatic grade 3 prolongation in QTc interval (observed after dose reduction to 90 mg/day), and febrile neutropenia were observed in 1 patient each. The 2 patients who experienced pancytopenia and QTc prolongation withdrew from the study. Following the occurrence of DLTs in 3 of the first 5 patients treated at the 135-mg quizartinib dose, the dose was reduced to 90 mg/day quizartinib for the next dosing cohort. There were no further DLTs reported at the 90-mg/day quizartinib dose; 90 mg/day was therefore considered the MTD, and a dose-expansion cohort was initiated. A total of 8 patients were enrolled at 90 mg/day before the study was closed to enrollment.

All patients in both dose groups experienced at least 1 treatment-emergent adverse event (TEAE). The most common TEAEs (occurring in ≥2 patients) are presented in Additional file 2. Most frequent treatment-related TEAEs were fatigue (*n* = 7), dysgeusia (*n* = 5), neutropenia (*n* = 3) and QTc prolongation (*n* = 3). Seven patients experienced treatment-related grade ≥ 3 TEAEs, the majority of which were hematologic. Grade 3 increases in QT corrected by Fridericia’s formula (QTcF) (defined as > 60 msec increase versus baseline) were observed in 4 of 8 patients in the 90-mg group and in 3 of 5 patients in the 135-mg group (Table 2). Of these, 2 patients in the 90-mg group and 1 in the 135-mg group had medical histories of cardiovascular disease.

**Table 2:** Summary of QTc prolongations (safety population)

	Treatment group, 90 mg (*n* = 8)	Treatment group, 135 mg (*n* = 5)	Total *N* = 13
Maximum value, *n*			
≤ 450 msec	2	3	5
> 450 to ≤480 msec	4	1	5
> 480 to ≤500 msec	1	0	1
> 500 msec	1	1	2
Maximum change from baseline, *n*			
≤ 30 msec	2	0	2
> 30 to ≤60 msec	2	2	4
> 60 msec	4	3	7

*QTc* corrected QT interval

Two patients in each dose group experienced SAEs (hematologic AEs, infections, and GI disorders), 3 of which were considered related to the study drug (Table 3). Of the 3 patients with hematologic SAEs, 1 patient received transfusions while 1 other patient received both transfusions and growth factor treatment. There were no deaths during or within 30 days of treatment discontinuation. Twelve patients were alive at follow-up (42–90 days after the first dose of quizartinib).

**Table 3:** Treatment-emergent serious adverse events (safety population)

Serious adverse events (SAEs)	Treatment group, 90 mg (*n* = 8)	Treatment group, 135 mg (*n* = 5)	Total (*N* = 13)
Patients with any SAE *n* (%)	2 (25.0)	2 (40.0)	4 (30.8)
Anemia	1 (12.5)	0	1 (7.7)
Febrile neutropenia	0	1 (20.0)	1 (7.7)
Leukopenia	1 (12.5)	0	1 (7.7)
Pancytopenia	0	1 (20.0)	1 (7.7)
Thrombocytopenia	1 (12.5)	0	1 (7.7)
Pneumonia	1 (12.5)	0	1 (7.7)
Urosepsis	0	1 (20.0)	1 (7.7)
Peritoneal hemorrhage	1 (12.5)	0	1 (7.7)

The same patient may have experienced more than 1 SAE

### Efficacy and PK/PD results

**Fig. 1:**
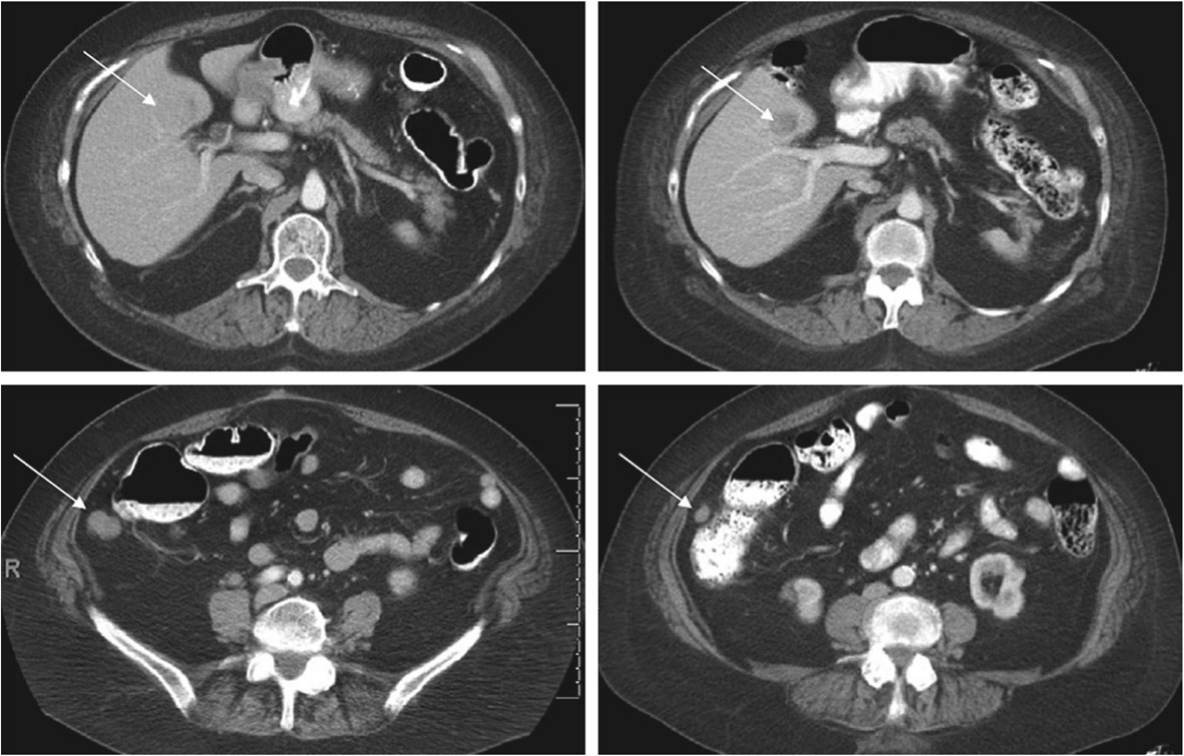
Tumor response with quizartinib monotherapy in a patient with GIST. Computed tomography scans of a patient with GIST demonstrated a 27% reduction in tumor burden with quizartinib monotherapy at the end of Cycle 1. Panels on the left represent baseline scans; panels on the right are from end of Cycle 1. *GIST* gastrointestinal stromal tumor

There were no complete or partial responses in the study. Six patients (46.2%) had a best response of stable disease, including 3 patients with GIST (all in the 90-mg dose group; all of whom had progressed on prior imatinib therapy), 1 patient with colorectal cancer (90 mg), 1 patient with sarcoma (135 mg), and 1 patient with thyroid cancer (135 mg). Notably, 1 patient with *KIT* exon 9 mutant GIST tumor (Y503_F504insAY mutation) had a 27% reduction in tumor burden after cycle 1 (Fig. 1), but withdrew from the trial by choice due to persistent GI symptoms before the follow-up evaluation. Pharmacodynamic analyses were not performed because of the small sample size. As a result, levels of inhibition of KIT or PDGFRA with quizartinib treatment could not be established. Pharmacokinetic analyses were not conducted because of the small sample size of completed data. Quizartinib PK has been characterized and reported in an earlier phase 1 study in patients with AML [23].

## Discussion

This study was designed to establish the MTD and tolerability profile of quizartinib, and to gain preliminary evidence of antitumor activity in solid malignancies, in a patient population enriched for diseases whose pathophysiology is related to aberrant signaling through KIT or PDGFRA such as GIST, other sarcomas or mucosal melanomas. Grade 3 dose-limiting toxicities of pancytopenia, QTc prolongation, and febrile neutropenia were observed in 3 of the first 5 patients enrolled in the starting 135-mg/day dose group. As a result, the next dose level was 90 mg/day, wherein no additional DLTs were observed. The safety profile of quizartinib was generally consistent with previous experience in AML studies at these doses of quizartinib [24, 25].

The small sample sizes in each dose group precluded quantitative assessment of the relationship between quizartinib dosing and incidence of AEs. Nonetheless, the observed AEs in this study were as expected in heavily pretreated patients, including the potential effects of longstanding impact from prior therapy on treatment tolerability. For example, cumulative myelosuppression after multiple prior regimens of cytotoxic chemotherapy could have exacerbated quizartinib-induced cytopenias. The most frequent AEs were hematologic, which is consistent with the known activity of quizartinib against myeloid progenitor cells. Results from this study may better characterize quizartinib’s safety profile.

This study establishes the MTD of quizartinib in heavily pretreated patients with advanced solid tumors at 90 mg/day. This MTD is consistent with the 60-mg dose of quizartinib currently under investigation as monotherapy in R/R AML [28]. Although we were unable to evaluate the effect of quizartinib on the activity of potential target kinases (eg, KIT and PDGFR) because of the small sample size, the lack of objective response to quizartinib in this study might suggest that the MTD does not adequately inhibit KIT/PDGFR. This is in contrast to the experience in patients with R/R *FLT3*-ITD AML, where lower doses of quizartinib have revealed effective kinase inhibition and demonstrated that quizartinib monotherapy at a target dose of 60 mg is clinically efficacious and has reduced toxicity risk, consistent with quizartinib selectivity and potency against FLT3 [26]. Although no PK was assessed in this study due to limited sample size, it has been characterized in phase 1 study in patients with AML [23]. A dose-dependent increase in the systemic exposure of quizartinib and its active metabolite AC886 was observed in the tested range of 12–450 mg [23].

## Conclusions

Quizartinib demonstrated limited evidence of antitumor activity as monotherapy at its MTD in this small phase 1 study. Although 9 of 13 patients in our study had eventual disease progression, stable disease was observed in 6 patients (all of whom had disease progression on multiple prior therapies). Disease stabilization in all 3 patients with GIST suggests that patients with advanced solid tumors who have progressed following treatment with RTK inhibitors may benefit from switching to a structurally distinct KIT inhibitor. This possibility is supported by a recent study wherein dovitinib, a multikinase inhibitor, demonstrated a clinically meaningful benefit when administered to patients with imatinib-refractory GIST [29]. Although no further studies of quizartinib in patients with solid tumors are planned at this time, the potential activity of quizartinib against tumors with established dependence on aberrant RTK activity (eg, KIT and PDGFR) or in a targeted population with *FLT3*-ITD mutations cannot be ruled out. Presently, development of quizartinib is focused around hematologic malignancies. Additional research is needed to establish the quizartinib doses needed to effectively inhibit KIT, PDGFRA, and PDGFRB RTKs and to evaluate the feasibility of administering these doses in the relevant patients.

## Additional files

Additional file 1: Scheduled assessments to evaluate the safety and tolerability of quizartinib. (DOCX 18 kb)

Additional file 2: Most common (reported in ≥2 patients) treatment-emergent adverse events (safety population). (DOCX 18 kb)

## Abbreviations

AE: Adverse event
AML: Acute myeloid leukemia
CSF1R: Colony-stimulating factor 1 receptor
CYP3A: Cytochrome P450 3A
DLT: Dose-limiting toxicity
ECG: Electrocardiogram
ECOG: Eastern Cooperative Oncology Group
FLT3: FMS-like tyrosine kinase 3
GI: Gastrointestinal
GIST: Gastrointestinal stromal tumor
ITD: Internal tandem duplication
LVEF: Left ventricular ejection fraction
MTD: Maximum tolerated dose
NCI CTCAE: National Cancer Institute Common Terminology Criteria for Adverse Events
PDGFR: Platelet-derived growth factor receptor
PK: Pharmacokinetics
QTc: Corrected QT interval
QTcF: QT per Fridericia’s Correction Formula
R/R: Relapsed/Refractory
RECIST: Response Evaluation Criteria in Solid Tumors
RTK: Receptor tyrosine kinase
SAE: Serious adverse event
SD: Standard deviation
T4: Thyroxine
TEAE: Treatment-emergent adverse event
TKI: Tyrosine kinase inhibitor
TSH: Thyroid-stimulating hormone
