# Supplementary material for: Safety and tolerability of quizartinib, a FLT3 inhibitor, in advanced solid tumors: a phase 1 dose-escalation trial
Source: BMC Cancer. 2018 Aug 6;18:790. doi: 10.1186/s12885-018-4692-z (PMC6080548; doi:10.1186/s12885-018-4692-z)
Supplement: Supplementary file 1 — Scheduled assessments to evaluate the safety and tolerability of quizartinib. (DOCX 18 kb) [file 12885_2018_4692_MOESM1_ESM.docx]

Additional Files

**Additional File 1** Scheduled assessments to evaluate the safety and tolerability of quizartinib

|  | Cycle 1 | | | | | Cycle 2 | | Cycle 3 | Ongoing visits every 13–15 days after Day 1 of current or subsequent cycle | Ongoing visits every 25-31 days after Day 1 of current or subsequent cycle | Early termination visit–within 6–8 days after last dose | Follow-up within 30 days after last dose |
| --- | --- | --- | --- | --- | --- | --- | --- | --- | --- | --- | --- | --- |
|  | Day 1 | Day 2 | Day 8 | Days 14–16 | Day 16 | Day 1 | Day 15 | Day 1; End of cycle 2 |  |  |  |  |
| Physical examination | x |  | x | x |  | x | x | x | x | x | x |  |
| Vital signs | x |  | x | x |  | x | x | x | x | x | x |  |
| Determination of ECOG performance status | x |  |  | x |  | x | x | x | x | x | x |  |
| AE/SAE assessment | x | x | x | x | x | x | x | x | x | x | x | x |
| 12-lead ECGs | x |  | x | x |  | x | x | x |  | x |  |  |
| Collection of plasma samples | x | x | x | x | x | x | x | x |  | x | x |  |
| Collection of blood and plasma samples | x | x | x | x |  | x |  |  |  |  | x |  |
| Clinical laboratory tests (chemistry, hematology, urinalysis) | x |  | x | x |  | x | x | x | x | x | x |  |
| Determination of free T4 and TSH |  |  |  |  |  | x |  | x |  | x |  |  |
| *AE* adverse event, *ECG* electrocardiogram, *ECOG* Eastern Cooperative Oncology Group, *SAE* serious adverse event, *T4* thyroxine, *TSH* thyroid-stimulating hormone | | | | | | | | | | | | |
