# Supplementary material for: Safety and tolerability of quizartinib, a FLT3 inhibitor, in advanced solid tumors: a phase 1 dose-escalation trial
Source: BMC Cancer. 2018 Aug 6;18:790. doi: 10.1186/s12885-018-4692-z (PMC6080548; doi:10.1186/s12885-018-4692-z)
Supplement: Supplementary file 2 — Most common (reported in ≥2 patients) treatment-emergent adverse events (safety population). (DOCX 18 kb) [file 12885_2018_4692_MOESM2_ESM.docx]

Additional Files

**Additional File 2** Most common (reported in ≥ 2 patients) treatment-emergent adverse events (safety population)

|  | Treatment group,  90 mg  (*n* = 8) | Treatment group, 135 mg  (*n* = 5) | Total  (*N* = 13) |
| --- | --- | --- | --- |
| Any TEAE, n (%) | 8 (100.0) | 5 (100.0) | 13 (100.0) |
| Fatigue | 5 (62.5) | 3 (60.0) | 8 (61.5) |
| Dysgeusia | 2 (25.0) | 3 (60.0) | 5 (38.5) |
| Nausea | 3 (37.5) | 1 (20.0) | 4 (30.8) |
| Vomiting | 2 (25.0) | 2 (40.0) | 4 (30.8) |
| Edema peripheral | 2 (25.0) | 1 (20.0) | 3 (23.1) |
| Peripheral sensory neuropathy | 1 (12.5) | 2 (40.0) | 3 (23.1) |
| Anemia | 2 (25.0) | 1 (20.0) | 3 (23.1) |
| Neutropenia | 2 (25.0) | 1 (20.0) | 3 (23.1) |
| Electrocardiogram QT prolonged | 1 (12.5) | 2 (40.0) | 3 (23.1) |
| Tumor pain | 1 (12.5) | 2 (40.0) | 3 (23.1) |
| Abdominal distension | 1 (12.5) | 1 (20.0) | 2 (15.4) |
| Dizziness | 0 | 2 (40.0) | 2 (15.4) |
| Leukopenia | 1 (12.5) | 1 (20.0) | 2 (15.4) |
| Thrombocytopenia | 1 (12.5) | 1 (20.0) | 2 (15.4) |
| Decreased appetite | 0 | 2 (40.0) | 2 (15.4) |
| Hyperglycemia | 2 (25.0) | 0 | 2 (15.4) |
| Dyspnea | 1 (12.5) | 1 (20.0) | 2 (15.4) |
| Pain in extremity | 1 (12.5) | 1 (20.0) | 2 (15.4) |
| Treatment-emergent adverse events (TEAEs) are those adverse events (AEs) with onset dates on or after the first dose of study drug, up through 30 days after the last dose, or those AEs that begin prior to the first dose of study drug and worsen in severity.  If a patient experienced more than 1 AE within a system organ class, the patient is counted once under that system organ class.  If a patient had more than 1 event for a particular preferred term, the patient was counted once under that preferred term | | | |
